# Model and experiences of initiating collaboration with traditional healers in validation of ethnomedicines for HIV/AIDS in Namibia

**DOI:** 10.1186/1746-4269-5-30

**Published:** 2009-10-23

**Authors:** Kazhila C Chinsembu

**Affiliations:** 1 Department of Biological Sciences, Faculty of Science, University of Namibia, P/B 13301, Windhoek, Namibia

## Abstract

Many people with Human Immunodeficiency Virus/Acquired Immunodeficiency Syndrome (HIV/AIDS) in Namibia have access to antiretroviral drugs but some still use traditional medicines to treat opportunistic infections and offset side-effects from antiretroviral medication. Namibia has a rich biodiversity of indigenous plants that could contain novel anti-HIV agents. However, such medicinal plants have not been identified and properly documented. Various ethnomedicines used to treat HIV/AIDS opportunistic infections have not been scientifically validated for safety and efficacy. These limitations are mostly attributable to the lack of collaboration between biomedical scientists and traditional healers. This paper presents a five-step contextual model for initiating collaboration with Namibian traditional healers in order that candidate plants that may contain novel anti-HIV agents are identified, and traditional medicines used to treat HIV/AIDS opportunistic infections are subjected to scientific validation. The model includes key structures and processes used to initiate collaboration with traditional healers in Namibia; namely, the National Biosciences Forum, a steering committee with the University of Namibia (UNAM) as the focal point, a study tour to Zambia and South Africa where other collaborative frameworks were examined, commemorations of the African Traditional Medicine Day (ATMD), and consultations with stakeholders in north-eastern Namibia. Experiences from these structures and processes are discussed. All traditional healers in north-eastern Namibia were willing to collaborate with UNAM in order that their traditional medicines could be subjected to scientific validation. The current study provides a framework for future collaboration with traditional healers and the selection of candidate anti-HIV medicinal plants and ethnomedicines for scientific testing in Namibia.

## Background

Namibia has a generalized Human Immunodeficiency Virus/Acquired Immunodeficiency Syndrome (HIV/AIDS) epidemic, with 17.8% of antenatal clinic attendees being HIV positive [1]. With over 230,000 people, out of a total population of about 2 million, living with HIV/AIDS, Namibia is one of the top five HIV/AIDS-affected countries in the world [2,3]. By March 2007, there were about 41,000 Namibians on antiretroviral treatment (ART) [4]. The number of people on ART increased to about 70,000 by August 2009.

Despite this impressive progress, Chinsembu [5] cautioned that Namibia's ART programme is like a candle in the wind as it battles to glimmer against the inevitable possibility of dying from another form of AIDS- 'Acquired Income Deficiency Syndrome'. There are concerns that the country's free public sector ART programme is not sustainable due its heavy reliance on donor funds. Besides funding, access to treatment in Namibia will have to cross many rivers including: lack of confidentiality, lack of bed space, lack of transport to hospitals, shortages of qualified health workers, long queues, the criterion of treatment supporter, and side-effects now causing new forms of stigma [5].

Many HIV-infected Namibians have access to antiretroviral drugs, but some still use traditional medicines to treat opportunistic infections and offset side-effects from antiretroviral medication. In the case of rural Namibian communities, formal biomedical services are also hardly accessible. Thus whilst the majority of Namibian HIV/AIDS patients rely on the current ART programme, some still have faith in traditional medicines. In general, HIV/AIDS patients are vulnerable in their choice of treatments, such that they can vacillate from conventional ART programmes to traditional healers and vice versa; they want to have the best of both worlds.

The inclusion of traditional medicines in official HIV/AIDS policy is an extremely sensitive and contentious issue in Southern Africa, the epicenter of HIV/AIDS. It is sensitive because traditional medicines can easily become a scapegoat for denial and inertia to roll-out ART as was the case during President Thabo Mbeki's South Africa. It is also contentious because in many resource-poor settings in Sub-Saharan Africa, government-sponsored ART programmes discourage the use of traditional medicines, fearing that the efficacy of antiretroviral drugs may be inhibited by traditional medicines, or that their interactions could lead to toxicity [6]. Reliance on traditional medicines can also lead to a discontinuation of ART therapy [7]. Thus many African governments still have contradictory attitudes towards traditional medicines for AIDS, discouraging it within ART programmes, and supporting it within their initiatives of public health and primary health care.

Prior to the availability of free and cheaper generic antiretroviral drugs, the World Health Organization (WHO) recommended that traditional healers be included in national responses to HIV/AIDS [8]. In this light, African governments expressed a need for a concerted, systematic and sustained effort at both local and regional levels to support and validate African traditional medicines on several fronts, including evaluation of traditional remedies, spiritual aspects of healing, HIV prevention and care, standardization of processing and packaging traditional remedies, as well as protection of indigenous knowledge and intellectual property rights [9]. The importance of investing in the high growth sectors of biotechnology and phytomedicine was also articulated in the founding document of the New Partnership for Africa's Development (NEPAD), and later adopted by the African Biosciences Initiative [10,11].

To popularize this commitment, the Organization of African Unity (African Union) Heads of State and Government declared the period 2000-2010 as the Decade of African Traditional Medicine. In addition, the Director General of WHO, declared 31^st ^August of every year as the African Traditional Medicine Day (ATMD) [8]. Further to these declarations, the Eastern and Southern Africa Regional Initiative on Traditional Medicine and AIDS convened a consultative meeting in May 2003 [8]. All these initiatives demonstrate the need to mainstream and institutionalize traditional medicine into the formal health care system. Puckree and others [12] emphasized that health care professionals need to be proactive in integrating traditional healing with westernized medicines in order to promote health for all.

Although there are a good number of reports on traditional uses of plants to treat various diseases, knowledge on herbal remedies used to manage HIV/AIDS is scanty, impressionistic and not well documented [13]. One known exception is Tanzania, where a detailed ethnobotanical study documented the status and use of traditional medicines in the management of HIV/AIDS opportunistic infections [14]. Besides the problem of lack of documentation, two other barriers continue to circumvent the integration of traditional medicines with modern conventional medicines.

The first barrier is that traditional remedies have neither been rigorously evaluated nor properly standardized. They are also poorly prepared, packaged and preserved. These drawbacks limit the use of traditional medicines for the treatment of HIV/AIDS [13]. The second obstacle speaks to how collaboration between traditional healers and biomedical scientists can be initiated, operationalized, and sustained. Here, the concern is how to initiate collaboration between two health care systems that differ in theory of disease causation and management [13].

In spite of the above dilemma, collaboration is essential, given the changing epidemic of HIV and the dynamic relationship between the two health sectors [15]. Thus experts recommend that health care providers must open lines of communication with traditional healers [16]. However, initiating collaboration is not as easy it appears to be from the literature, and if it is to be meaningful, systematic steps must be followed in order to arrive at sustainable collaboration between traditional healers and biomedical scientists [13]. Previous attempts at collaboration with the traditional health care sector have evolved around organizing traditional healers into national associations, training programmes for healers, and laboratory testing of herbs [17]. Kayombo and co-workers [13] found that effective collaboration should involve signing Memoranda of Understanding (MOU) covering intentions, obligations and mutual responsibilities of all parties. The MOU should also outline how benefits ensuing from the collaboration will be distributed [13].

There are renewed attempts to open communication with traditional healers in Namibia. These efforts will help biomedical practitioners gain useful insights into the work of traditional healers. However, this does not mean that traditional healers are now part of the country's strategy against HIV/AIDS. The spirit to seek collaboration of traditional healers is not a point of departure from ART. In fact, there are no reliable scientific data regarding the use of traditional medicines for the management of HIV/AIDS in Namibia. Within this prism of caution, it is clear that the only way to improve the quality and prolong the life of Namibian HIV/AIDS patients is through the use of available antiretroviral drugs.

On the other hand, the current use of generic antiretroviral drugs still faces several challenges including, HIV resistance, toxicity, limited availability, and lack of curative effect [18]. Thus it is important to search for novel anti-HIV agents which can expand the current arsenal against HIV. Natural sources, particularly plants, appear a promising source of medicines for symptomatic treatment of Sexually Transmitted Diseases (STDs) including AIDS (see review by Vermani and Garg [19]). In the past two decades, a substantial amount of research has been done worldwide, and a lot more is in progress, to isolate anti-HIV active leads from plants.

Indigenous knowledge [20], coupled with a history of safe use and ethnopharmacological efficacy [18,19], present a faster approach to discover new anti-HIV agents. This new approach is now being called reverse pharmacology [20]. Following this approach, a novel class of integrase inhibitors, the dicaffeoylquinic acids (DCQAs), was isolated from medicinal plants in Bolivia [21,22]. Since Namibia has a rich biodiversity and a long tradition of medicinal use of plants, there is potential to isolate novel chemical compounds that can inhibit key HIV enzymes. Such plant-based compounds could improve the efficacy of existing drugs and retard the emergence of drug-resistant HIV [21].

Some Namibian traditional healers could be living libraries or reservoirs of indigenous knowledge and insightful experiences that could open new vistas in the identification of local plants with novel anti-HIV activity. Such genuine traditional healers should be carefully selected, on a case-by-case basis, because some traditional healers are charlatans. Given such a benefit of a doubt, some Namibian traditional medicines could present possible leads for the identification of novel anti-HIV compounds.

Despite the potential to isolate novel anti-HIV agents from Namibian medicinal plants, there has been little engagement of traditional healers by the scientific community. To-date, no collaborative effort between Namibian healers and scientists has been initiated and no agreements to work together are in place. As a result of this lack of operative collaboration, bioprospecting for local medicinal plants with novel anti-HIV compounds has not been done, and traditional medicines used by healers to treat HIV/AIDS opportunistic infections have not been scientifically validated.

Responding to the compelling need for evidence regarding traditional medicines, NEPAD and Southern African Network for Biosciences (SANBio) launched a flagship project to validate traditional medicines for the affordable treatment of HIV/AIDS and opportunistic infections. NEPAD/SANBio would like to replicate this flagship project to many other countries. However, scientific researchers in many countries have been unable to find the entry point into the sphere of traditional medicines because of the various institutional and intellectual property rights' bottlenecks that impede access to traditional healers and their medicines.

This article presents a five-step model of initiating collaboration with Namibian traditional healers so that local plants and ethnomedicines that may contain novel anti-HIV compounds are identified and subjected to scientific validation. Further to the model, the article describes and discusses experiences from the five steps or processes that were used to initiate collaboration, namely the: (i) National Biosciences Forum; (ii) multi-stakeholder steering committee with the University of Namibia (UNAM) as the focal point; (iii) study tour to institutions in Zambia and South Africa where validation of traditional medicines for HIV/AIDS was being undertaken; (iv) commemoration of the ATMD; and (v) consultations with various stakeholders in the project area.

## Research Methodology

This study used a five-step research methodology:

1. In order to initiate research on traditional medicines, the Ministry of Education's Directorate of Research, Science and Technology (ME-DRST) organized a transparent and participatory National Biosciences Forum (NBF) on November 20, 2007. Local stakeholders including the academia, government departments, and Non-Governmental Organizations (NGOs) attended the NBF. Officials from NEPAD/SANBio and the Council for Scientific and Industrial Research (CSIR) laboratories, Pretoria, South Africa, also attended the forum, and presented keynote papers on collaborative initiatives involving the validation of ethnomedicines used by traditional healers to treat HIV/AIDS.

2. The NBF formed a multi-stakeholder steering committee to spearhead the validation of traditional medicines in Namibia. The NBF also unanimously elected UNAM as focal point of the steering committee and mandated the institution to initiate research on the validation of traditional medicines for affordable treatment of HIV/AIDS and opportunistic infections in Namibia.

3. In July 2008, a one-week study tour was undertaken to Zambia (Ministry of Health and Sondashi clinic), and to the CSIR laboratories in Pretoria, South Africa. A co-chairperson from the focal point of the steering committee and the chairperson of the Namibia Traditional Healers Association conducted the study visit. The study tour was important because the Ministry of Health in Zambia was collaborating with CSIR laboratories in validating a herbal formula used by Ludwig Sondashi, a traditional healer from Zambia, to treat HIV/AIDS.

4. Commemorations of the ATMD allowed UNAM to gain access to individual traditional healers and other stakeholders. Two elaborate lines of communication led to the successful relationship between the UNAM focal point and the Ministry of Health. First, the Permanent Secretary (PS) of the Ministry of Health, Windhoek, requested the UNAM Pro-Vice Chancellor for Academic Affairs and Research (PVC-AAR) to nominate a representative to sit on the national committee organizing the commemoration of the ATMD. The PVC-AAR then nominated the focal point to represent UNAM on the ATMD national organizing committee. Second, the PS had also agreed to an invitation by the PVC-AAR to nominate a Ministry of Health representative to the steering committee.

5. Stakeholders in the study area (north-eastern Namibia) were informed about the research. These included government officials (regional health directors, hospital superintendents, primary health care administrators, regional health administrators, community forestry officers, traditional leaders, chairpersons of traditional healers' associations, and key informants such as teachers and church leaders. Opinions of these stakeholders towards traditional healers were ascertained during unstructured interviews as recommended by Agar [23]. The stakeholders were also requested to furnish for 'leads' to local traditional healers. Finally, an exploratory and descriptive case study of traditional healers was conducted in four administrative regions of north-eastern Namibia: Kavango, Omusati, Oshana, and Caprivi. Data collected during the exploratory and descriptive case study are not included in the present article.

## Results

### Five step model used to initiate collaboration with traditional healers in Namibia

**Figure 1:**
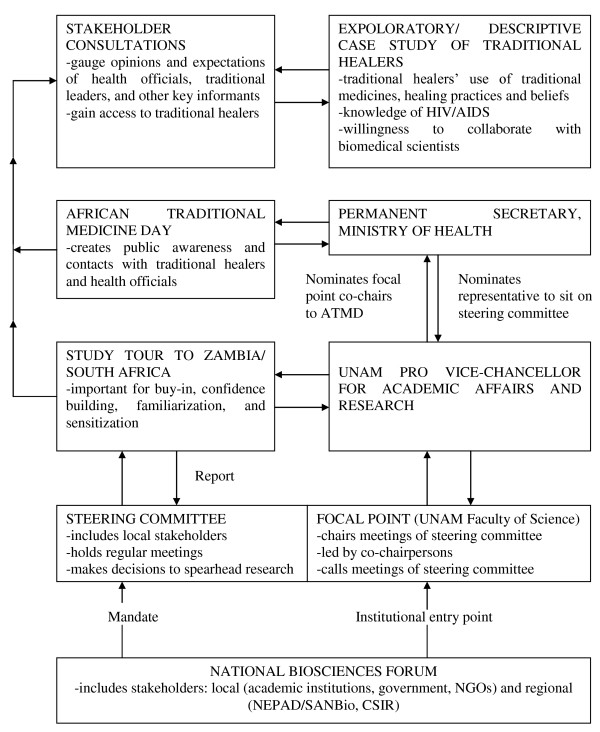
**Five step model for initiating collaboration with traditional healers in Namibia**.

Figure 1 presents the five-step contextual model used to initiate collaboration with traditional healers in Namibia. The flow chart starts with the NBF (step 1), the structure that established the steering committee (step 2a) and mandated UNAM as the focal point (step 2b), to spearhead the selection of candidate anti-HIV medicinal plants and validation of traditional medicines used to treat HIV/AIDS and related opportunistic infections. The study tour (step 3) enabled the steering committee and the focal point to gain invaluable insights into various collaborative frameworks with traditional healers, and the scientific validation process. Through the organization and commemoration of the ATMD (step 4), and consultations with stakeholders in north-eastern Namibia (step 5a), UNAM made contacts with and gained access to traditional healers, who where then interviewed in an exploratory and descriptive case study (step 5b). All the traditional healers were eager to subject their ethnomedicines to scientific validation, and were willing to collaborate with UNAM in that regard. This Chinsembu model was successful in initiating collaboration between UNAM and traditional healers in Namibia.

### Experiences from the five distinct processes of the model

#### National Biosciences Forum

The NBF was the 'seed' administrative structure that established a multi-stakeholder steering committee mandated to guide research towards the validation of traditional medicines for the treatment of HIV/AIDS and opportunistic infections in Namibia. UNAM did not have a national institutional mandate to study traditional medicines for treatment of HIV/AIDS and opportunistic infections in Namibia. Through the multi-stakeholder consensus and mandate derived from the NBF, UNAM (Faculty of Science) found an institutional entry point to commence work on the validation of traditional medicines for HIV/AIDS treatment.

#### Steering committee and UNAM focal point

The role of UNAM as the focal point was to call and chair meetings of the steering committee, whose other members were: Polytechnic of Namibia; Namibia Traditional Healers Association; Ministry of Agriculture, Water and Forestry (Directorates of Research and Forestry); ME-DRST; Ministry of Health and Social Services; Namibia Chamber of Commerce and Industry; Health Professionals Councils of Namibia; three NGOs namely, Namibia Network of AIDS Service Organizations (NANASO), the Regional Agricultural and Environment Initiatives Network-Africa (RAEIN-Africa), and the Centre for Research Information Action in Africa- Southern African Development and Consulting (CRIAA-SADC); National Botanical Research Institute (NBRI), and the Indigenous Plant Task Team (IPTT).

Steering committee meetings helped to gauge stakeholder expectations and anxieties. For example, certain members of the steering committee were against the idea of setting up a national pharmacopeia of plants used to treat HIV/AIDS and related opportunistic infections. They were also critical of UNAM's linkages with NEPAD/SANBio and CSIR. Such criticisms were often centred on the controversial issues of biopiracy, intellectual property rights, and benefit sharing. A study tour to Zambia and South Africa was undertaken in order to find out how the two countries had dealt with such sensitive issues.

#### Study tour to Zambia and South Africa

For a long time, many traditional healers in Zambia claimed 'cures' for HIV/AIDS. However, these claims had not been subjected to scientific validation. In November 2005, an open observational and exploratory clinical trial to validate traditional medicines used to treat HIV/AIDS started in Lusaka. The objective of the trial was to evaluate for efficacy of the formulations, and to establish change in the physical and clinical status of HIV positive volunteers. Three herbal formulations (Sondashi, Mayeyanini, and Mailasine) were tested in 12, 11 and 9 HIV-positive adult volunteers, respectively. It was not known whether the volunteers had previously been on antiretroviral treatment. However, their CD4 counts were determined at the onset of the trial. The formulations were also tested for any spicing with conventional antiretroviral drugs. The trial ended in April 2006.

Results of that trial showed that all the three formulations had anti-HIV activity, as measured by an increase in CD4 counts and reduction in viral loads, and did not have significant liver and renal toxicity (Patrick Chikusu, personal communication). Of the three formulations, the Sondashi formula was found to be the best candidate for further validation. One of the volunteers in the clinical trial was interviewed by the visiting Namibian researchers; he confirmed that the Sondashi formula increased his CD4 count and reduced the viral load. He also extolled how the Sondashi formula had restored his health.

The Sondashi herbal formula was invented by Ludwig Sondashi, a former government minister and constitutional lawyer with a PhD. He had first used it to treat his sick son. At that time, Dr. Sondashi tried several herbs but his son died. He then combined four plants that he used to treat relatives with HIV/AIDS. He discovered that the herbs were treating HIV/AIDS-related opportunistic infections.

The Zambian researchers led by Patrick Chikusu recommended that further studies of the Sondashi formula (SF-2000) be carried out using a larger group of volunteers, and for a longer period of time. To further validate the Sondashi formulation, NEPAD/SANBio took the herbal formulation for further tests at the CSIR laboratories in Pretoria, South Africa. Isolation of novel anti-HIV agents from SF-2000 is being undertaken at the CSIR.

CSIR had legal frameworks that guide collaboration with indigenous knowledge holders and traditional healers, including heads of agreements with clauses on commercialization and benefit sharing, publications, patents, and formation of databases. In South Africa, the government issued a bioprospecting permit only when a benefit sharing agreement was in place. Such benefits were usually given to communities, led by trust committees formed by traditional healers. CSIR entered into agreements with trust committees, or with individual traditional healers, provided any such trust committees were informed. Thus the principle of prior informed consent was followed.

During bioprospecting, traditional healers were paid money to offset costs incurred during plant collection. Such payments did not constitute sale of plant materials, otherwise traditional healers would forfeit future benefits. Sample delivery notes were used whenever plant materials were handed over to the CSIR. According to CSIR collaborative frameworks, a patent was a publication, and traditional healers were not part of the patent. However, traditional healers were entitled to benefits before patenting.

Validation of the Sondashi formula at the CSIR was a unique challenge because the collaborative process involved many stakeholders from another country- Zambia. The process was also complicated by the trans-boundary shipment of plant materials. A different model of collaboration was therefore used to circumscribe these challenges. A consortium of institutional stakeholders, managed by a steering committee, which included a lawyer, entered into different agreements such as the consortium agreement encompassing all stakeholders, memorandum of agreement with the individual traditional healer, and materials transfer agreement with the government.

#### African Traditional Medicine Day

Commemoration of the sixth ATMD in August 2008 was the first of its kind in Namibia. Meetings of the organizing committee were held at the Ministry of Health headquarters in Windhoek. The meetings provided an important forum for the chairpersons of the steering committee to interact with traditional healers and other stakeholders. Members of the organizing committee were informed about the new initiative to validate traditional medicines for the treatment of HIV/AIDS and opportunistic infections.

The ATMD also helped to create public awareness of traditional medicines in Namibia. Public awareness campaigns were conducted through television, radio, newspapers, exhibitions of traditional medicines, displays of publications on traditional medicines, and releases of press statements. Under the theme "the role of traditional health practitioners in primary health care", celebrations of the ATMD provided the public with a rare glimpse into the work of traditional healers. Many traditional healers also felt that they had an important contribution to public health. Official commemorations of the sixth ATMD were held in Windhoek, the capital city of Namibia, while those for the seventh ATMD in August 2009 were held in Rundu, Kavango region. During the Rundu celebrations, traditional healers exhibited their ethnomedicines, and explained to the public the medicinal properties of the various plants. The author presented a keynote paper covering findings of the exploratory and descriptive case study of traditional healers in Namibia. The presentation was televised on the Namibia Broadcasting Corporation main news bulletin of September 7, 2009.

#### Stakeholder opinions of traditional healers in north-eastern Namibia

Stakeholders in north-eastern Namibia had various opinions about traditional healers in general, and validation of ethnomedicines used for HIV/AIDS treatment, in particular. Health officials such as the regional health directors and primary health care administrators were negative about traditional healers. One primary health care official stated that she "did not trust these people". However, 90% of stakeholders indicated their willingness to work with UNAM to validate traditional healers' ethnomedicines because "it was necessary to throw out the bad herbs and promote the good ones". They were also keen to learn about the outcome of the scientific validation of the Sondashi formula from Zambia. Community leaders such as headmen and regional health administrators were more positive about traditional healers in their communities. They formed the best entry points to traditional healers, who were eager to collaborate with UNAM and other stakeholders in order that their ethnomedicines could be scientifically validated.

## Discussion

King [24] reviewed the conceptual elements necessary to initiate collaboration between traditional healers and biomedical scientists in the management of HIV/AIDS in Africa. Several country-specific models of collaboration between biomedical and traditional health practitioners in the field of HIV/AIDS management were discussed. Kayombo and others [13] scrutinized King's conceptual elements of collaboration and examined their practical appropriateness in initiating collaboration in Tanzania. This study presents a collaborative model that works in Namibia's context. Models for controlled clinical evaluation of traditional medicines for HIV/AIDS treatment exist [25] but such models do not adequately address the initial but critical stages of collaboration between biomedical scientists and traditional healers. The protocols pay more attention to clinical practice than the administrative structures that precede the validation process. The current contextual model describes distinct administrative structures and processes that addressed the unique challenges found at the interface between biomedical science and traditional medicine in Namibia. This is important because UNAIDS best practice models have shown that sensitivity to the political, environmental, cultural and economic contexts is crucial to the success of collaborative interventions [26]. The model helps to establish collaboration with traditional healers so that candidate plants that may contain novel anti-HIV agents are identified and traditional medicines used to treat HIV/AIDS opportunistic infections are subjected to scientific testing.

Lebeau [27] studied the practice of traditional medicine in Namibia, but as early as 1993, WHO had already tried to partner with traditional healers in the management of HIV/AIDS [28]. By 1996, traditional healers were registered by the Traditional Healers Council of Namibia, established in March 1996, under the Allied Health Services Professions Act of 1993 (Act 20 of 1993). The Traditional Healers Council was mandated to register traditional healers and instill professionalism in their trade. In 2004, the Allied Health Services Professions Act of 1993 was replaced by the Allied Health Professions Act (Act no. 7 of 2004), and the Traditional Healers Council was subsequently dissolved. A Traditional Healers Bill was then proposed but it is still under discussion. These dynamics have left many Namibian traditional healers in a legal quagmire and lacuna.

However, the dissolution of the Traditional Healers Council was not just a legal technicality or coincidence. It had been a long time coming, as government authorities were seemingly getting impatient with traditional healers that claimed to cure HIV/AIDS, and turned the epidemic into a cash-cow. This was no different to the "AIDS opportunism" and "AIDS entrepreneurship" described by Richter [29]. There were also public protests against media reports of HIV-infected adults that had sex with minors, ostensibly because they were instructed by their traditional healers that sex with virgins would cure HIV/AIDS [30]. Namibian traditional healers had not only managed to turn the HIV/AIDS epidemic into a profitable enterprise but also a moral minefield. The practice of traditional medicine in Namibia assumed a tarnished public image.

Research institutions willing to initiate collaboration with traditional healers would therefore have to tread very carefully, because these developments brought stigma to genuine traditional healers, who retreated into treating their patients at night, and created flashpoints of conflict with their biomedical counterparts. Richter [29] confirmed that there was friction between biomedicine and ethnomedicine in South Africa, often because biomedical scientists were frustrated with traditional healers for claiming to cure HIV/AIDS, and failing to refer their HIV positive clients to the hospital, not until their immune systems had completely broken down.

Traditional healers were seen to be preventing the access of HIV/AIDS patients to conventional antiretroviral drugs, brought into the country under the United States of America President's Emergency Programme for AIDS Relief (PEPFAR) and Global Fund. The work of traditional healers was seen as being antagonistic to the rollout of antiretroviral drugs; this would obviously invite the intervention of government officials and donors, who are usually defensive in the face of criticism [31]. Against this background, Namibian traditional healers treating HIV/AIDS-related conditions were isolated, thus it was very difficult to initiate collaboration with those that would have wanted to subject their ethnomedicines to scientific validation.

It would appear, therefore, that the Traditional Healers Council was abolished for five reasons: (a) charlatan traditional healers that claimed AIDS cures, (b) public outrage against traditional healers that encouraged sex with minors as a cure for AIDS, (c) lack of referral of HIV/AIDS patients to hospitals, (d) possible friction with donors regarding conventional ART programmes, and (e) fears of non-compliance to conventional ART programmes. Despite the current legislative lacuna regarding Namibian traditional health practitioners, the model presented in this study forms a sympathetic and respectful effort to engage with Namibian traditional healers.

There are other reasons that make the initiation of collaboration with traditional healers a very sensitive issue. Namibia is the driest African country south of the Sahara. Therefore, it is not surprising that traditional communities and authorities are concerned about the management of the country's natural resources. Falk [32] stated that customary norms control access to resources, limit their extraction, regulate the technologies of resource use, and prescribe clear consequences for non-compliant behaviour. The sustainable use of Namibia's biodiversity is also enshrined in the country's constitution. Article 95 of the Namibian constitution states that: "the state shall actively promote and maintain the welfare of the people by adapting policies aimed at... the maintenance of ecosystems, essential ecological processes and biological diversity of Namibia and utilization of living natural resources on a sustainable basis, for the benefit of all Namibians, both present and future" [33].

Namibia is a diamondiferous country but biodiversity, and medicinal plants in particular, are now considered to be the country's 'green diamonds'. Ironically, Article 95 of the Namibian constitution has not been translated into subsidiary laws to govern bioprospecting, and access and benefit sharing. Government has instituted the National Biodiversity Programme (NBF), the IPTT, and the Interim Plant Bioprospecting Council (IPBC), mandated by Cabinet to deal with matters of indigenous plants and knowledge, genetic resources, access and benefit sharing (ABS). A Bill on ABS has been drafted but technical questions remain unanswered [34]. Even if UNAM had applied to the Ministry of Environment for a research permit, approval of such an application would have been probably stranded in the prevailing legislative vacuum.

Despite the lack of specific and enforceable legislation related to bioprospecting and ABS, government has realized the value of the country's medicinal plants. As was well argued by Reihling [35], such a realization stems from the fact that trade in medicinal plants forms a 'hidden economy' that supports self-dependent plant gatherers, street vendors and healers, and forms part of income generating strategies of rural households. Yet Namibia is also home to market-exchange high value medicinal plants such as the Devil's claw. By 1981, Namibia (then South West Africa as it was called under Apartheid South African rule), was known to export 200 tons per year of Devil's claw [36]. Exports of Devil's claw were estimated at 600 tons a year in 1998 [37], and in excess of 1000 tons in 2002 [38]. Ever since Namibian plants such as the Devil's claw and *Hoodia cactus *entered the speculative marketplace of biocapital [35], the country's political leadership has become alive to the intrigues of scientific validation and commercialization of indigenous medicinal plants.

Speaking at a symposium on the Devil's claw in 2001, Namibia's first President, H.E. Sam Nujoma, said: "I believe that while scientific research is necessary to improve the way in which our natural resources are exploited...our people must not be completely disowned... of resources that they have possessed for generations. It will be a sad day when the medicinal formulas of Devil's claw are patented by big pharmaceutical companies and thereby become depleted and unavailable to the natural owners of the resource" [37]. Still, such statements have not helped to put money into the pockets of the local people.

Despite the end of Apartheid in 1990, the country's indigenous people have not benefited from the lucrative biotrade in the Devil's claw, whose sales in Germany soared to 30 million Euros as of 2001 [39]. A purified extract from the Devil's claw was patented by Finkelberg, a leading Germany company [35], and as President Sam Nujoma had feared, the originators of the plant were neither addressed through patent negotiations nor did they benefit from the large commercial success of the drug in Germany where it turned into the third most frequently used natural drug of all [35]. A 50 gram extract of Devil's claw in tablet form fetched 25 Euros in 2007 [35] but Namibian gatherers were paid 30-40 cents per kilogram of dried tubers [40].

The case of the Devil's claw demonstrates that Namibians have had little benefit from bioprospecting and commercialization of their medicinal plants. It is within this prism of economic dispossession that the entry point for research into traditional medicines in Namibia was confronted by moral and economic persuasions. Thus the scientific validation of traditional medicines used for HIV/AIDS had to be initiated in a very responsible and transparent manner. There was a looming danger that UNAM researchers would be seen to be mediating and colluding with the interests of foreign commercial entities and capital. To avoid such pitfalls, UNAM had to tread carefully. It was important for UNAM to strike an understanding with capable authorizing institutions, thus the NBF provided a transparent mechanism because it brought together all stakeholders from government ministries, watchdog institutions such as IPBC and IPTT, academia, and NGOs. Elsewhere, the role of NGOs was found to be crucial in determining the success or failure of collaboration [41,42].

For ethical reasons, officials from the IPBC were not part of the steering committee. In meetings of the steering committee, it became apparent that issues of intellectual property rights, indigenous knowledge, ownership, benefit sharing, biopiracy, trade secrets, materials transfer and confidentiality contracts would cloud, and if not properly handled, scuttle UNAM's efforts to collaborate with traditional healers in order to identify plants with novel anti-HIV compounds, or validate ethnomedicines used to treat HIV/AIDS opportunistic infections. There were questions about UNAM's links with the CSIR, which had previously patented P57, the active ingredient from *Hoodia *[35]. Later, CSIR granted a license to exploit the patent to Phytopharm, a UK company [35]. In 2003, an ABS contract granted the San people 6% share of the financial benefits received by the CSIR from its private partners. However, it is believed that the *Hoodia *ABS contract had not benefitted the Namibian San [34].

While UNAM may have genuine interests in pursuing collaborations that could lead to the development of novel anti-HIV medicines for the public good, Chokshi [43] in the United States of America described cases where Universities which owned patents for antiretroviral drugs (stavudine, emtricitabine, and tenofovir) had sold the intellectual property rights for the medicines to private pharmaceutical industries. The steering committee also agreed to set up a Namibian pharmacopeia of traditional plants used to treat HIV/AIDS and related opportunistic infections. This would help preserve knowledge about prospective ethnomedicines and indigenous plants with novel anti-HIV activity. This is vital given that most healers were old and dying with their libraries of knowledge. Similar documentation efforts were conducted in Tanzania [14], though the idea to create a database or to publish such data in the public domain remains a contentious issue.

Namibian traditional healers and the chairperson of their association were not present at the NBF. However, since chairpersons of traditional healers' associations are important gatekeepers, it was noted that the success of the Namibian validation initiative would largely depend on the strategic engagement of the Namibian Traditional Healers Association. It was envisaged that collaboration with Namibian traditional healers would depend on how tactfully the steering committee engages with the chairperson of the Namibian Traditional Healers' Association. Given that initiation of collaboration was a new effort in Namibia, a lot of suspicions were bound to cloud the process. Therefore, it was clear that a process of buy-in, confidence building and learning would have to be carried out before traditional healers agree to collaborate in the identification of anti-HIV medicinal plants or the scientific testing of their traditional herbal formulations.

The sensitization, fact-finding and familiarization study tour to Zambia and South Africa by the co-chairperson of the steering committee and the chairperson of the Namibian Traditional Healers Association helped to achieve the above objectives and set the stage for collaboration. During the visit, the Namibian team obtained first-hand information from the various stakeholders in Zambia and CSIR. By learning what their Zambian counterparts had done, the Namibian Traditional Healers' Association developed the confidence to initiate collaboration with UNAM. The study visit helped to dispel mistrust and open a new chapter of collaboration to work towards a mutual goal.

In Tanzania, researchers found that leaders of traditional healers' associations appeared to be important gatekeepers that can open or close the door to other healers willing to collaborate with biomedical scientists [13]. Such leaders of traditional healers often have reservations based on questions surrounding the sharing of benefits from the intended collaboration. Kayombo and co-workers [13] are unequivocal on the point that leaders of traditional healers' associations and other influential opinion leaders are gateways to traditional healers, and thus the need to treat them very carefully lest one cannot access genuine traditional healers for collaboration. It is important that consultations with leaders of traditional healers' associations should cultivate trust, confidence and respect. In reality, the process to bring traditional healers on board can be arduous and long. While the collaborative framework from Tanzania provides useful lessons, there exist unique challenges in every country.

Apart from informing the collaborative process in Namibia, the study tour also shed light on the importance of co-existence between traditional and conventional health care. For example, the success of the open observational and exploratory clinical trial in Zambia was attributed to the joint collaborative effort arising from the good working relationship with traditional healers [44]. The good working relationship was a deliberate initiative from government, which made the chairperson of Traditional Health Practitioners' Association of Zambia (THPAZ) become a member of the Sector Advisory Group (which monitors health sector activities in Zambia), the traditional medicine research committee, and the Central Board of Health. The involvement of traditional healers' associations into mainstream health sector planning and implementation helped to create trust, an important ingredient which formed the cornerstone of the collaboration between traditional healers and biomedical scientists in Zambia. The clinical trial was also guided by a clear MOU which dealt with issues of intellectual property rights and benefit sharing.

Further, the sustainability of the collaboration in validating the SF-2000 herbal formulation was also underlined by the fact that in Zambia, the Ministry of Health had made tremendous progress in integrating traditional medicines into the health care delivery system. Health care was delivered in a parallel approach, with traditional and conventional medicines running side by side. Traditional healers were allowed to operate their own clinics. Thus, although conventional antiretroviral drugs were the standard form of HIV/AIDS treatment, the government did not take the use of traditional medicines to be in conflict. However, the interactive effects of combining western and traditional medicines have not been investigated. For example, it was not clear whether promotion of traditional medicines for HIV/AIDS would reduce adherence to and efficacy of conventional ART. Government was also cautious of dubious traditional healers that claimed to cure HIV/AIDS. A fake cure for AIDS called Tetrasil was marketed by a Zambian newspaper editor in conjunction with a United States of America AIDS denialist. Tetrasil was later found to be a pesticide used to clean swimming pools [45].

Therefore, the Ministry of Health regulated all research into traditional medicines in Zambia. To that end, government in January 2008 produced guidelines for research in traditional medicines [46]. The guidelines were adapted from those of the WHO. Further, the Ministry of Health devised the Traditional Medicine Practice Council Bill, and the Traditional Healers Practitioners Policy. These instruments were aimed at regulating the practice of individual traditional healers, and the various traditional healers' associations, notably the Traditional Health Practitioners' Association of Zambia, Zambia National Council of 'Ngangas', and the Zambia Herbalist United Organization.

At the CSIR, the Biosciences Research Programme was involved in bioprospecting and drug discovery. The programme was premised on a theoretical framework linking biodiversity, science and indigenous knowledge, an important ingredient known to accelerate drug discovery through reverse pharmacology [20]. Indigenous knowledge was found to reduce the timeframe, from therapeutic concept to phase I clinical trial, from 15 to 3 years. The CSIR had state-of-the-art scientific equipment for the identification of novel anti-HIV compounds and for scientific validation of traditional medicines. These included a spray drier used to make crude herbal extracts following the principles of good manufacturing practice. The study tour therefore helped the Namibian visitors to appreciate the scientific and technical realities of the validation process, and that it was expensive. A similar study tour to Ghanaian institutions was undertaken by researchers of the Malawian steering/technical committee that begun work on traditional medicines [47].

In my contextual collaborative model, the fourth process involves celebrations of the African Traditional Medicine Day. This process is a strong link that helps to popularize the use of traditional medicines, apart from changing the public image of traditional healers. The celebrations allow interactions among government and WHO officials, traditional healers and the steering committee. Thus commemorations of the ATMD form a unique gateway to traditional healers. This is important because it is a sign that government is changing its negative attitude towards traditional healers. The recognition of traditional healers will also leverage pressure on Parliament to enact the law on traditional medicine.

Participation of the stakeholders is critical to the ownership of the whole process of validation of traditional medicines. Hence the research team had to explain the project to local stakeholders. We found that traditional leaders were a very useful lead to traditional healers in their communities. The involvement of community leaders also gave the researchers the confidence that they were dealing with genuine traditional healers. Since traditional leaders command the respect of their subjects, it was very easy for traditional healers to open up to the research team in the company of their community leaders. In Tanzania, studies have shown that community leaders are important in selecting competent and trustworthy traditional healers for collaboration [13].

On the other hand, health officials were skeptical about traditional medicines. They only began to soften their stance upon being told by the research team that most pharmaceutical drugs were actually derived from indigenous plants and knowledge. Except for the Chief Health Programme Administrator in Rundu, and the Regional HIV/AIDS Director in Katima Mulilo, most health officials did not know any traditional healers in their communities, hence they were not a good entry point to traditional healers. The negative attitude of health officials in north-eastern Namibia is not conducive for sustainable collaboration. Western trained officials, especially medical doctors, seem to downplay the role of traditional healers [13]. This shows that despite the new spirit to collaborate with traditional healers, there is still need for educational programmes to change the mindset of health officials in north-eastern Namibia.

The attitude of these skeptical health officials can be explained by the fact that the work of traditional healers is not watched by a professional body. The training of traditional healers and the quality of their treatment are not professionally controlled. Further, the work of diviners is not easy to comprehend. While the work of herbalists is much easier to understand since they select and apply plant remedies, the work of diviners that diagnose diseases using the 'ndaula' (beads) or those that deal with 'mulaleka' borders on special spiritual powers sometimes called witchcraft. 'Mulaleka' is a sexual practice founded in witchcraft. It is believed that someone can have sex by remote, with someone that lives in another place, often without consent. 'Mulaleka' was originally meant to assist men to have sex with their wives that lived in faraway places, especially husbands that had immigrated to cities in search of jobs. However, the practice was now widely used by men to engage in forced sexual intercourse with women that they admired but have not consented to their sexual proposals. Women who refused sexual proposals from men often became victims of 'mulaleka'. Sometimes older men also engaged in 'mulaleka' with younger women and girls. It is widely believed that HIV-infected men that practice 'mulaleka' also spread AIDS. Traditional healers in the Caprivi region treated women to protect them from 'mulaleka'. Herbs were applied in wounds after bleeding patients around the waist. Plant roots were also put under the pillow during sleep to protect would-be victims of 'mulaleka'.

In the Caprivi region, the distinction between herbalists and diviners has become blurred. Many traditional healers are both herbalists and diviners, treating the patient's physical and spiritual needs. With an incurable condition like AIDS, the spiritual aspects of treatment could be very important [29]. Yet, to many health officials, it is the diviners that make the profession of traditional medicine look very suspicious and bogus. This is especially so because, during Apartheid rule, the work of diviners was prohibited under the Witchcraft Suppression Act of 1957 and the Witchcraft Suppression Amendment Act of 1970. It seems that the mindset of Namibian biomedical practitioners and society that resent traditional healers is still stuck in the past laws of Apartheid.

Since traditional healers are the first point of call for many patients in north-eastern Namibia, it is important that both traditional healers and western trained health officials find common ground of collaboration. Synergy between western trained doctors and traditional healers will improve health care delivery in Namibia. For example, traditional healers could learn best practices from western trained doctors; this could help reduce infections. In South Africa, it was found that traditional healers had a crucial role to play in building the health care system, including strengthening and supporting national responses to HIV/AIDS [29]. Indeed, many trained doctors now recognize traditional healers as potential allies in the fight against HIV/AIDS [48].

Many traditional healers were willing to collaborate with biomedical practitioners, with a view of subjecting their ethnomedicines to scientific validation. However, their eagerness to collaborate should be taken cautiously, as it could be driven by commercial motives and the desire for recognition and reputation. Already, there are complaints that traditional healers are charging exorbitant fees for their services. During the ATMD celebrations, a traditional leader (Hompa) in Rundu, Kavango region, castigated traditional healers who demand cows from dying patients. Profit-seeking motives and untrustworthiness of traditional healers could jeopardize future collaboration with biomedical scientists.

## Conclusion

The model presented in this study shows that deliberate steps can initiate collaboration between biomedical researchers and traditional healers. In settings where there is no legislation on bioprospecting and benefit sharing, and where research institutions have no prior national mandate to initiate work on scientific validation of traditional medicines, participatory forums of stakeholders, including potential authorizing bodies and government watchdog committees, should be constituted to ensure a transparent and responsible research ethic. In this study, the National Biosciences Forum created a transparent entry point for UNAM to initiate collaborative efforts towards validation of traditional medicines used for HIV/AIDS treatment. To further enhance transparency, a steering committee of stakeholders can guide initiation of collaboration with traditional healers. Members of such a steering committee should be willing to accept criticisms from one another, and learn from other countries. Scientific validation of traditional medicines is not a cheap process. Therefore, research institutions from other countries should form part of the collaborating consortium, provided specific legal agreements are in place. Fears of biopiracy and inequitable sharing of benefits should not overwhelm the genuineness and urgency of collaborative attempts to scientifically validate traditional medicines for the greater public good. Local avenues that enhance interaction between traditional healers and biomedical scientists should also be exploited. Such interactions will largely depend on the strategic engagement of stakeholders by the research institution. Community leaders, not health officials, were found to be important gateways to traditional healers. Initialization of collaboration with traditional healers will enable biomedical scientists to identify indigenous plants that appear to be promising sources of medicines for symptomatic treatment of STDs including AIDS. It will also lead to the scientific testing of various ethnomedicines that traditional healers apply. The five-step model is therefore a preliminary but important prerequisite to the next aspect of the project: identification of candidate plants and scientific testing of traditional medicines in Namibia.

## Competing interests

The author declares that they have no competing interests.

## Authors' contributions

KC developed the contextual model, spearheaded the research project and collaboration with traditional healers, and wrote the manuscript.

## References

[B1] Government of the Republic of Namibia (2008). Report on the 2008 National HIV Sentinel Survey: HIV prevalence rate in pregnant women, bi-annual survey 1992-2008.

[B2] Government of the Republic of Namibia (2002). The National Strategic Plan on HIV/AIDS. Third medium term plan 2004-2009.

[B3] USAID (2006). USAID Namibia website.

[B4] Katjitae I Update on the Namibian ART programme. Paper presented at the Namibian HIV Clinicians' Society Annual Conference held at Safari Court Hotel, Windhoek, Namibia, 5-9 March 2007.

[B5] Chinsembu KC (2007). Access to treatment still has many rivers to cross. Report presented to Panos Southern Africa Regional HIV/AIDS Programme, Lusaka, Zambia.

[B6] Hardon A, Desclaux A, Egrot M, Simon E, Micollier E, Kyakuwa M (2008). Alternative medicines for AIDS in resource-poor settings: insights from exploratory anthropological studies in Asia and Africa. J Ethnobiology and Ethnomedicine.

[B7] Langlois-Klassen D, Kipp W, Jhangri GS, Rubaale T (2007). Use of traditional herbal medicine by AIDS patients in Kabarole District, western Uganda. Am J Trop Med Hyg.

[B8] Homsy J, King R, Tenywa J, Kyeyune P, Opio A, Balaba D (2004). Defining minimum standards of practice for incorporating African traditional medicine into HIV/AIDS prevention, care and support: a regional initiative in Eastern and Southern Africa. J Altern Complement Med.

[B9] UNAIDS (1998). The regional workshop on adopting minimum standards of practice for THETA evaluation team. Participatory evaluation report. Innovation or reawakening? Roles of traditional healers in the management and prevention of HIV/AIDS in Uganda.

[B10] NEPAD (2001). The New Partnership for Africa's Development Founding document. Abuja, Nigeria.

[B11] African Biosciences Initiative (2005). Business Plan 2005-2010.

[B12] Puckree T, Mkhize M, Mgobhozi Z, Lin J (2002). African traditional healers: what health care professionals need to know. Int J Rehabil Res.

[B13] Kayombo EJ, Uiso FC, Mbwambo ZH, Mahunnah RL, Moshi MJ, Mgonda YH (2007). Experience of initiating collaboration of traditional healers in managing HIV and AIDS in Tanzania. J Ethnobiology & Ethnomedicine.

[B14] Kisangau DP, Lyaruu HVM, Hosea KM, Joseph CC (2007). Use of traditional medicines in the management of HIV/AIDS opportunistic infections in Tanzania: a case in the Bukoba rural district. J Ethnobiology & Ethnomedicine.

[B15] Mills E, Singh S, Wilson K, Peters E, Onia R, Kanfer I (2006). The challenges of involving traditional healers in HIV/AIDS care. Int J STD AIDS.

[B16] Banda Y, Chapman V, Goldenberg RL, Stringer JS, Culhane JF, Sinkala M, Vermund SH, Chi BH (2007). Use of traditional medicine among pregnant women in Lusaka, Zambia. J Altern Complement Med.

[B17] King R (2005). Collaboration with traditional healers on prevention and care in sub-Saharan Africa: a practical guideline for programs.

[B18] Klos M, Venter M Van de, Milne PJ, Traore HN, Meyer D, Ooshuizen V (2009). *In vitro *anti-HIV activity of five selected South African medicinal plant extracts. J Ethnopharmacology.

[B19] Vermani K, Garg S (2002). Herbal medicines for sexually transmitted diseases and AIDS. J Ethnopharmacology.

[B20] Kaya HO, Kalua FA, Awotedu A, Kamwanja LA, Saka JDK (2009). Indigenous knowledge (IK) and innovation systems for public health in Africa. Science, technology and innovation for public health in Africa.

[B21] Zhu K, Cordeiro ML, Atienza J, Robinson WE, Chow SA (1999). Irreversible inhibition of Human Immunodeficiency Virus Type 1 integrase by dicaffeoylquinic acids. J Virology.

[B22] Robnison WE, Reinecke MG, Abdel-Malek S, Jia Q, Chow SA (1996). Inhibitors of HIV-1 replication that inhibits HIV integrase. Proc Natl Acad Sci USA.

[B23] Agar AH (1980). The professional stranger: an informal introduction to ethnography.

[B24] King R (2000). Collaboration with traditional healers in HIV/AIDS prevention and care in sub-Saharan Africa: a literature review. UNAIDS Best Practice Collection.

[B25] Chaudhury RR (2001). A clinical protocol for the study of traditional medicine and human immunodeficiency virus-related illness. J Alternative Complimentary Med.

[B26] King R (2006). Collaborating with traditional healers for HIV prevention and care in sub-Saharan Africa: suggestions for programme managers and field workers. UNAIDS Best Practice Collection.

[B27] Lebeau D (1998). Urban patients' utilization of traditional medicine: upholding culture and tradition.

[B28] Boadu SO (1993). Involving traditional healers in the AIDS campaign: is true partnership possible?. Int Conf AIDS, WHO/GPA, NACP, Windhoek, Namibia.

[B29] Richter M Traditional medicines and traditional healers in South Africa. Discussion paper prepared for the Treatment Action Campaign and AIDS Law Project, 27 November 2003.

[B30] Ahmad K (2001). Namibian government to prosecute healers. The Lancet.

[B31] Kamwi R, Kenyon T, Newton G (2006). PEPFAR and HIV prevention in Africa. The Lancet.

[B32] Falk T (2009). Biodiversity and the ancestors: challenges to customary and environmental law. Namibian Law Journal.

[B33] Barnard P, Shikongo ST (2000). Namibia's national report to the fifth Conference of Parties on implementation of the Conference on Biological Diversity. Nairobi, Kenya.

[B34] Du Plessis P (2007). Indigenous knowledge and biotrade. Presentation at the National Biosciences Forum and validation of traditional medicines workshop, Safari Hotel, Windhoek.

[B35] Reihling HCW (2008). Bioprospecting the African renaissance: the new value of muthi in South Africa. J Ethnobiology & Ethnomedicine.

[B36] Rukangira E (2009). Medicinal plants and traditional medicine in Africa: constraints and challenges. Sustainable Development International.

[B37] Wickham L (2009). Devil's claw- the herbal solution to the north-south divide?. PositiveHealthOnline.

[B38] Mwandemele OD, Mshigeni K, Kosina P, Kamburona C (2006). Challenges of domesticating wild plants: the case of the Devil's claw (Harpagophytum spp.) in the Kalahari desert ecosystem. Discovery & Innovation.

[B39] Stewart KM, Cole D (2005). The commercial harvest of Devil's claw (Hypagophytum spp.) in Southern Africa: the devil's in the details. J Ethnopharmacology.

[B40] Wegener T (2000). Devil's claw: from African traditional remedy to modern analgesic and anti-inflammatory. HerbalGram.

[B41] Berlin B, Berlin EA (2005). Community autonomy and the Maya ICBG project in Chiapas, Mexico: How a bioprospecting project that should have succeeded failed. Human Organization.

[B42] Rosenthal J (2006). Politics, culture, and governance in the development of prior informed consent in indigenous communities. Cultural Anthropology.

[B43] Chokshi DA (2006). Improving access to medicines in poor countries: the role of Universities. PLoS Medicine.

[B44] Burnett A, Baggaley R, Ndovi-MacMillan M, Sulwe J, Hang'omba B, Bennett J (1999). Caring for people with HIV in Zambia: are traditional healers and formal health workers willing to work together?. AIDS Care.

[B45] Amon JJ (2008). Dangerous medicines: unproven AIDS cures and counterfeit antiretroviral drugs. Globalization and Health.

[B46] Government of the Republic of Zambia (2008). Guidelines for research in traditional medicines in Zambia.

[B47] Nyirenda KK (2009). Traditional medicines, medicinal plants and HIV/AIDS: an overview of programmes and direction for Malawi. Presentation at the CSIR workshop on SF-2000 CSIR, Pretoria, South Africa.

[B48] Liverpool J, Alexander R, Johnson M, Ebba EK, Francis S, Liverpool C (2004). Western medicine and traditional healers: partners in the fight against HIV/AIDS. J Natl Med Assoc.

